# Empowering Home Health Aides: Impact of Education on Job Satisfaction and Perceived Value in Patient Care

**DOI:** 10.1093/geroni/igaf122.3242

**Published:** 2025-12-31

**Authors:** Sasha Vergez, Hannah Mitlak, Joanna Bryan, Margaret McDonald, Madeline Sterling

**Affiliations:** VNS Health, Bronx, New York, United States; Tufts University, Medford, Massachusetts, United States; Weill Cornell Medicine, New York, New York, United States; VNS Health, New York, New York, United States; Weill Cornell Medicine, New York, New York, United States

## Abstract

Home health aides (HHAs) play a critical role in patient care, yet their contributions are often overlooked, and high turnover rates warrant the need for intervention. This abstract examines HHAs experiences, attitudes toward their work, and perceptions of their overall value in patient care after receiving educational training on heart failure (HF), as a part of a randomized controlled trial - education only vs. education plus use of chat application with nurse managers. Overall, 102 HHAs participated in the study (mean age: 54 years, 98% women, 73% with >5 years experience). Job satisfaction was measured before and 90 days after the course. While the percentage of participants who agreed they were satisfied with their job increased, the change was not statistically significant. Interviews, conducted 45 days post-course with a sub-set of HHAs (64%), were thematically analyzed. HHAs found the training engaging and informed. Many reported applying their knowledge to improve patient care, educate patients, and support families. Participants valued the training professionally and personally, including enhancing their confidence in patient care. Additionally, HHAs noted improved communications and stronger patient-relationships. Many HHAs expressed a desire for additional learning opportunities. The study highlights the potential of educational programs to improve care for other chronic conditions, while simultaneously supporting and improving engagement of HHAs, enhancing workforce efficacy, job satisfaction and possibly patient outcomes. Continuous learning fosters positive attitudes toward professional development and can help retain HHAs, a profession with historically high turnover rates.

